# Assessment of genetic variability for grain nutrients from diverse regions: potential for wheat improvement

**DOI:** 10.1186/s40064-016-3586-2

**Published:** 2016-11-03

**Authors:** Anamika Pandey, Mohd Kamran Khan, Erdogan E. Hakki, George Thomas, Mehmet Hamurcu, Sait Gezgin, Ozge Gizlenci, Mahinur S. Akkaya

**Affiliations:** 1 Department of Soil Science and Plant Nutrition, University of Selcuk, 42250 Konya, Turkey; 2 Department of Molecular and Cellular Engineering, Sam Higginbottom Institute of Agriculture, Technology and Sciences, Allahabad, 211007 India; 3 Department of Biology, Middle East Technical University, 06800 Çankaya, Ankara Turkey; 4 Biotechnology Program, Department of Chemistry, Middle East Technical University, 06800 Çankaya, Ankara Turkey

**Keywords:** Bread wheat, Geographical origin, Grain protein content, Macronutrient, Micronutrient

## Abstract

**Background:**

A total of 150 bread wheat genotypes representing 121 Indian and 29 Turkish origin were screened for nutrient concentrations and grain protein content. Elemental and grain protein composition were studied by Inductively Coupled Plasma-Atomic Emission Spectrophotometer and LECO analyser, respectively. The study was performed to determine the variability in nutrient concentrations present in the collected wheat genetic material from two countries.

**Results:**

Several fold variations among genotypes existed for almost all the elements. Three major components of principal component analysis (PCA) revealed 60.8% variation among the genotypes. Nutrient variables segregated into two groups, one group containing all the macroelements except sulphur; and another cluster containing proteins and all the microelements except Zn and Mn. Pearson correlation analysis and heat-map were in accordance with each other determining strong positive association between P–K, Mn–Zn, Mg–S and Cu–protein content. Also, PCA and hierarchical grouping divided all the Indian and Turkish genotypes in two main clusters.

**Conclusions:**

Nutritional profile differentiated the genotypes from two countries into separate groups. However, some of the varieties were closely associated and indicated the success of global wheat exchange programs. While most of the correlations were in agreement with the previous studies, non-association of zinc with grain protein content directed towards its control by some other genetic factors. Some of the experimental wheat varieties with promising nutrient content have been suggested for future wheat advancement programs. Results obtained will be supportive for breeders involved in wheat biofortification programs, food industries and people relying on whole grain wheat products.

**Electronic supplementary material:**

The online version of this article (doi:10.1186/s40064-016-3586-2) contains supplementary material, which is available to authorized users.

## Background

Domestication and selective breeding progression significantly contributed to the depletion of nutritional content and in minimizing the genetic diversity of crops (Ladizinsky 1998). The great “Green Revolution” diverted farmers’ interest from legumes to cereals and consequently, the yields of cereal grains especially of wheat increased tremendously. However, in this pursuit of filling the bellies, the objective of enhancing the nutritional value of grains lagged behind (Welch and Graham 2004). An analysis of the nutritional value, especially micronutrients in the CIMMYT-developed germplasm revealed a declining trend in the micronutrient levels of the varieties (Monasterio and Graham 2000; Ortiz-Monasterio et al. 2007; Velu et al. 2014).

Currently, over two billion people suffer from ‘‘hidden hunger’’, a term used to describe deficiency of micronutrients (FAO 2013). Hence, after a successful green revolution, a thriving “nutritional revolution” is a prerequisite (White and Broadley 2009). Human requires approximately fifty-one nutrients in adequate amount, including both macro and micronutrients for nourished and healthy lives (Welch 2008). Today, nutrient deficiencies are not only common in developing countries where diet is mainly dependent on cereals, but it is also prevalent in developed countries (Diaz et al. 2003; Whatham et al. 2008). Iron and zinc deficiencies affect about 40 and 33%, of the world population, respectively (Paesano et al. 2012; Wessells and Brown 2012). While both iron and zinc are limiting in the diets of Indians, zinc is limiting in the diet of Turkish people (Cakmak et al. 1999; Akhtar 2013; Anand et al. 2014). Zn is essential for a healthy immune system, growth, wound healing, reproduction, fertility and sense of taste and smell, whereas Fe plays a central role in the transportation of oxygen around the body (Berg et al. 2002; Gibson 2006). In infants and young people, reduced Fe levels can lead to impaired growth and behavioural problems (Baker and Greer 2010).

Undoubtedly, among the cereals, wheat is an imperative resource of energy throughout the world (FAOSTAT 2008; http://faostat.fao.org). Whole grain products of wheat are suggested to efficiently utilize its element rich aleurone layer and thus, providing extended good fitness (Slavin 2004). Moreover, wheat is parallel to soybean crop in protein supply to human and livestock as it is a staple diet in less developed countries (Shewry 2000). Hence, dietary significance of wheat proteins should be properly appreciated and attempts should be made to improve it. Billions of people depend on wheat for fulfilling their nutritional prerequisites because of its wide agronomic flexibility, ease of storage and the types of foodstuffs prepared by its flour. In such scenario, augmentation of nutritional quality of wheat products through breeding approaches can provide a sustainable solution against diseases. Selection of wheat varieties with natural elevated mineral content can easily facilitate this strategy.

Biofortification (referring to plant breeding based strategies for enhancement of micronutrients in the edible parts of the plants/crops) has been suggested among the top five solutions to the problem of micronutrient malnutrition at the Copenhagen consensus (2008). Additionally, Genetic Biofortification is considered as a potential resolution to mineral malnutrition (Singh et al. 2005; Uauy et al. 2006). In 2005, EU Healthgrain Project has been initiated to exploit the nutritional components of cereal grains including wheat (van der Kamp et al. 2014). Under this project, Shewry (2009) screened 150 bread wheat lines along with some other wheat species and cereals for grain protein content and reported 12.9–19.9% variation in wholemeal. In 2006, a fundamental initiative was taken by Harvestplus for the estimation of genetic variability of element content in six crops and to facilitate varietal development in the breeding programs of these crops (Bouis 1996; Cakmak et al. 1996; Anglani 1998; Graham et al. 2001; Gregorio 2002; Welch and Graham 2004; Welch 2002; White and Broadley 2009) (http://www.harvestplus.org). Existence of a large variation in element and grain protein content in various wheat and its related species has been documented in numerous studies (Cakmak et al. 2004; Oury et al. 2006; Morgounov et al. 2007; Škrbić and Onjia 2007; Peleg et al. 2008; Shi et al. 2008; Ficco et al. 2009; Zhao et al. 2009; Chatzav et al. 2010; Zhao et al. 2011; Harmankaya et al. 2012). High variability is reported for both iron and zinc concentration in wild emmer wheat (Peleg et al. 2008; Gomez-Becerra et al. 2010), followed by durum wheat (Velu et al. 2011; Badakhshan et al. 2013) and lower levels of variability in bread wheat (Graham et al. 1999; Oury et al. 2006). However, still persistent efforts are necessary to search innovative wheat genetic resources augmented with essential minerals and protein.

India and Turkey are renowned for their major role in global wheat production holding second and tenth position in the world (FAO 2014). Detection of the varieties with balanced nutrient and protein content from these two countries can facilitate the wheat biofortification programs worldwide. The knowledge of nature and magnitude of genetic variability in the population is of immense value for planning efficient breeding programme to improve the yield potential of the genotypes. The selected wheat germplasm resources from different countries may be utilized in the future to attain more nutritionally rich wheat food. Therefore, in the present study an effort has been made to determine (1) genetic diversity for grain protein and macro/microelements in 121 Indian and 29 Turkish bread wheat varieties, (2) the correlations among grain nutrients and their association with protein content, (3) relationship between eco-geographical origin of involved bread wheat varieties and their nutrient concentrations, (4) to understand the level of crucial macroelements (Ca, K, Mg, Na, P and S), microelements (Cu, Zn, Fe and Mn) and protein content in Indian and Turkish bread wheat varieties.

## Results

### Genetic diversity for grain nutrients and protein composition of wheat genotypes

**Fig. 1:**
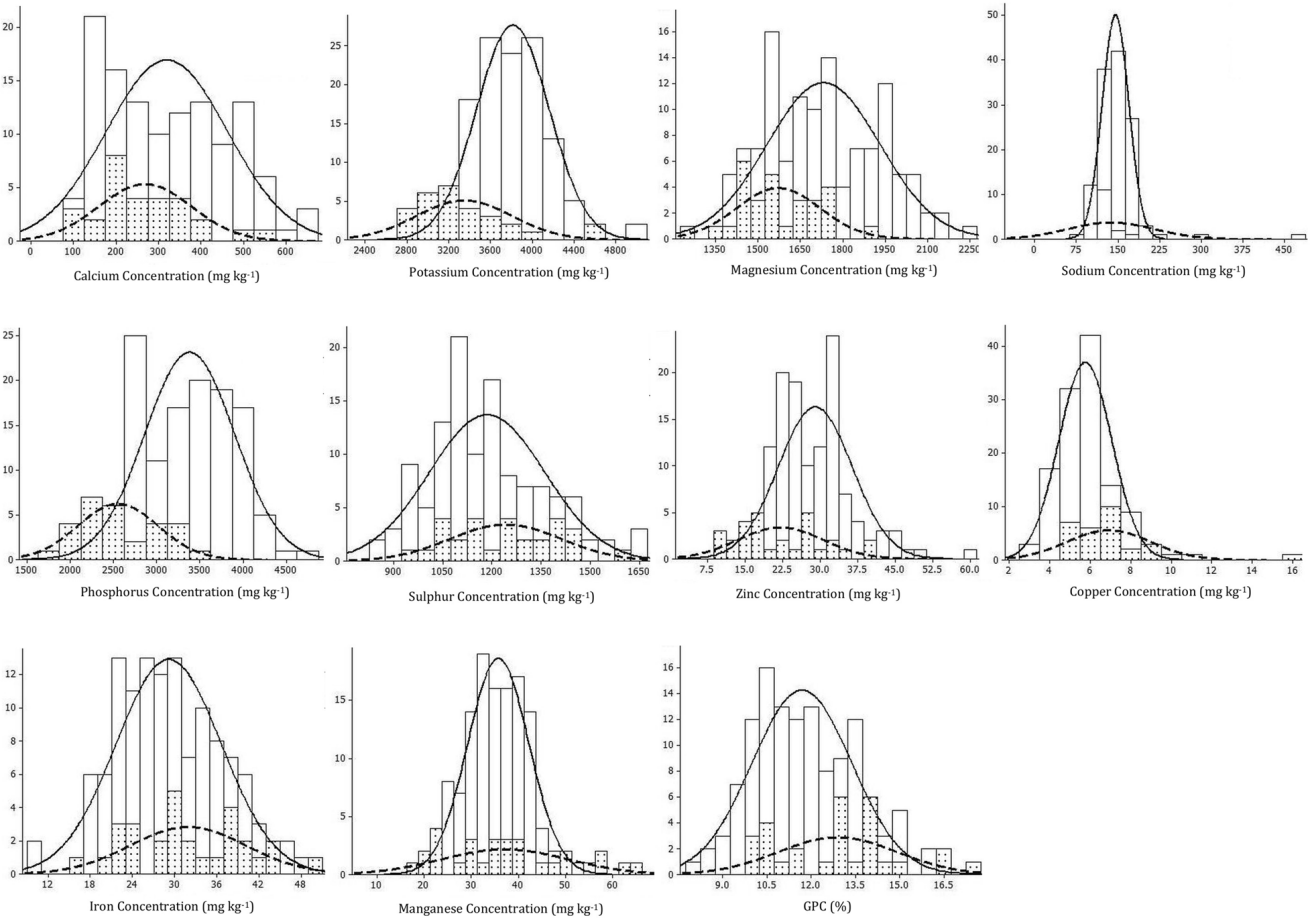
Frequency distribution of 121 Indian and 29 Turkish bread wheat genotypes for element concentrations and grain protein content. *Plain bars* represent distribution of Indian wheat genotypes while *dotted bars* denote Turkish wheat genotypes. *x-axis* denotes number of genotypes, while *y-axis* represents concentrations

Additional file 1: Table S1 presented the estimated concentrations of ten elements (calcium, potassium, magnesium, sodium, phosphorus, sulphur, zinc, copper, iron, manganese) and grain protein content in 150 Indian and Turkish wheat genotypes. In macro elements, grain Ca, K, Mg, Na, P and S content fluctuated by 6.4, 1.7, 1.8, 5.9, 2.7 and 2.0 times with a range varying from 104.3 to 663.5 mg/kg, 2834.2 to 4926.1 mg/kg, 1266.8 to 2251.1 mg/kg, 81.3 to 483.1 mg/kg, 1775 to 4720.3 mg/kg and 837.4 to 1673.1 mg/kg, respectively. Among microelements, grain Zn, Cu, Fe, Mn and protein content varied by 5.6, 4.7, 5.4, 3.6 and 2.2 fold ranging from 10.7 to 59.4 mg/kg, 3.3 to 15.6 mg/kg, 9.2 to 49.7 mg/kg, 18.1 to 65.6 mg/kg and from 8.0 to 17.3% correspondingly (Table 1). Indian and Turkish wheat genotypes showed normal frequency distributions for most of the elements except for Na and Cu, which did not distribute normally in Turkish genotypes (Fig. 1).

**Table 1:** Descriptive statistics of grain element and protein concentrations in the 150 bread wheat genotypes

Variable	Origin	Mean	SD	Min	Med	Max	Skewness	Kurtosis
Ca	INDIA	319.4	142.6	105.9	308.2	663.5	0.36	−0.93
	TURKEY	268.1	108.7	104.3	229.0	527.0	0.54	−0.14
K	INDIA	3814.3	349.7	2986.2	3789.3	4926.1	0.49	0.64
	TURKEY	3347.8	457.6	2834.2	3196.2	4597.5	1.44	2.06
Mg	INDIA	1732.0	200.0	1386.1	1721.2	2251.1	0.30	−0.73
	TURKEY	1572.2	147.2	1266.8	1542.8	1918.0	0.24	−0.13
Na	INDIA	145.5	24.1	85.3	141.9	235.4	0.44	0.95
	TURKEY	136.8	77.7	81.3	114.6	483.1	3.78	15.26
P	INDIA	3381.7	521.5	2227.2	3404.9	4720.3	0.10	−0.81
	TURKEY	2551.0	463.4	1775.0	2497.0	3472.5	0.30	−0.82
S	INDIA	1185.4	176.5	837.4	1165.3	1673.1	0.56	−0.01
	TURKEY	1242.1	169.6	929.1	1251.0	1623.8	0.11	−0.60
Zn	INDIA	29.0	7.4	16.7	28.3	59.4	1.07	1.85
	TURKEY	22.1	8.5	10.7	20.1	40.0	0.54	−0.62
Cu	INDIA	5.7	1.3	3.3	5.7	10.7	0.80	1.45
	TURKEY	6.9	2.1	4.5	6.8	15.6	2.66	10.46
Fe	INDIA	29.1	7.5	9.2	28.6	49.1	0.20	0.07
	TURKEY	31.9	8.2	15.6	30.5	49.7	0.12	−0.56
Mn	INDIA	35.7	6.5	22.5	35.3	58.6	0.49	0.75
	TURKEY	37.0	13.4	18.1	36.0	65.6	0.56	−0.45
GPC	INDIA	11.6	1.7	8.0	11.5	16.2	0.34	−0.39
	TURKEY	12.8	2.0	10.1	13.1	17.3	0.26	−0.50

Range of elements determined in our study was in accordance to that reported by Harmankaya et al. (2012) with Ca, K, Mg, Na, P and S contents of wheat varieties between 266 and 531 mg/kg, 3029 and 5566 mg/kg, 972 and 1525 mg/kg, 277 and 368 mg/kg, 2422 and 4610 mg/kg and 1241 and 2052 mg/kg, respectively. Additionally, wide range of Fe and Zn content reported in our experiments as 9.2–49.7 and 10.7–59.4 mg/kg were comparable to some previous studies with 24.2–43.1 and 10.4–38.2 mg/kg (Harmankaya et al. 2012), 21.3–30.6 and 14.9–19.3 mg/kg (Rawat et al. 2009), 27.3–41.9 and 16.1–27.2 mg/kg (Oury et al. 2006) and 28.8–50.8 and 13.5–34.5 mg/kg (Zhao et al. 2009), respectively.

Indian wheat genotypes revealed high average content for all the macroelements except Sulphur, while Turkish genotypes showed higher average content for all the microelements except zinc (Table 1). Average protein content was higher in Turkish genotypes (12.9%) as compared to Indian varieties (11.7%). Some of the Indian genotypes like DBW_77, HW_5202, K_8434, MACS_6222, UP_2511, UP_2696, Veeri and few Turkish varieties like Murat, Atilla, Demir were promising, rich in a number of macro and microelements as well as protein.

### Association between grain nutrients and protein content

**Fig. 2:**
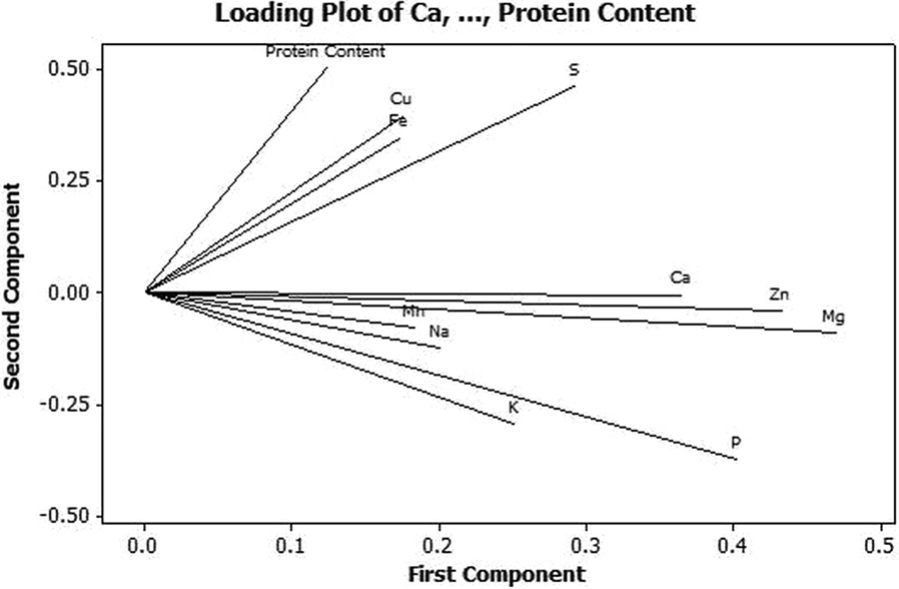
Principal component analysis loading plot based on correlation matrix of nutrients and grain protein content recorded on 121 Indian and 29 Turkish bread wheat genotypes. First principal component (PC1) explains 30.5% variation, while second principal component (PC2) describes 19.0% variation

Outcomes of the elemental and protein composition in 150 wheat genotypes were estimated using principal component analysis (PCA). Additional file 2: Table S2 represents the loading of all the elements and protein content on first three principal components and the variances elucidated by every component. PCA of all the Indian and Turkish wheat genotypes extracted three major principal components that collectively accounted for 60.8% variation. PC1 (Fig. 2; Additional file 2: Table S2) explained 30.5% variation and was positively loaded with all the elements and grain protein content (GPC). PC2 explained 19.0% variation and was positively loaded with sulphur, copper, iron, protein content and negatively loaded with calcium, potassium, magnesium, sodium, phosphorus, zinc and manganese. PC3 explained 11.4% variation and was positively loaded with K, Mg, Na, P, S and protein content. On PC1, positively loaded, Ca, Mg, P, Zn and on PC2, positively loaded S, Cu, Fe, protein and negatively loaded P were dominant variables, respectively.

Loading plot of first two principal components determined two main groupings of elements: first group was composed of all the macroelements except S along with two micronutrients, Zn and Mn; another group consisted of two microelements, Cu and Fe along with grain protein content and one macroelement, S (Fig. 2). This was in accordance with correlation analysis results that showed significant positive associations among several macroelements (Table 2). Mg showed positive correlation with Ca and K; Na with Ca, K and Mg and P with Ca, K, Mg and Na. In microelements, iron was moderately and highly positively correlated with zinc and copper, respectively. Another appealing feature was the strong considerable association of microelements with macroelements in line with the principal component analysis. Like, Zn allied with macroelements Ca, K, Mg, P; Mn associated with Ca, P and S linked with Cu and Fe. GPC was positively coupled with sulphur, copper and iron (Fig. 2).

**Table 2:** Coefficients of correlation (r) between concentrations of grain protein (GPC) and mineral nutrients in a collection of 150 bread wheat genotypes from India and Turkey

	Macro-elements						Micro-elements			
	Ca	K	Mg	Na	P	S	Zn	Cu	Fe	Mn
*Macro*-*elements*										
K	0.030									
p value	0.717									
Mg	0.615**	0.318**								
p value	0.000	0.000								
Na	0.224*	0.201*	0.293**							
p value	0.006	0.014	0.000							
P	0.324**	0.622**	0.738**	0.290**						
p value	0.000	0.000	0.000	0.000						
S	0.312**	0.056	0.387**	0.033	0.044					
p value	0.000	0.496	0.000	0.686	0.589					
*Micro*-*elements*										
Zn	0.492**	0.341**	0.544**	0.164*	0.538**	0.319**				
p value	0.000	0.000	0.000	0.045	0.000	0.000				
Cu	0.025	0.039	0.118	0.018	−0.059	0.433**	0.255*			
p value	0.763	0.632	0.151	0.824	0.472	0.000	0.002			
Fe	0.158	−0.084	0.202*	0.048	0.011	0.300**	0.172*	0.348**		
p value	0.053	0.309	0.013	0.560	0.891	0.000	0.036	0.000		
Mn	0.228*	−0.032	0.151	−0.061	0.263*	0.051	0.373**	0.147	0.049	
p value	0.005	0.694	0.065	0.461	0.001	0.536	0.000	0.073	0.551	
GPC	0.072	−0.076	0.101	0.043	−0.151	0.633**	0.099	0.270*	0.231*	−0.144
p value	0.381	0.357	0.220	0.603	0.065	0.000	0.227	0.001	0.004	0.079

Asterisks indicate significance at *p < 0.05 and at **p < 0.001)

It was interesting to find a constructive association of zinc and manganese with macroelements like Ca, K, Mg, P, Na and linkage of S with microelements like Cu and Fe (Fig. 2). In addition, sulphur was strongly associated with Zn and protein content.

### Association between geographical origin and grain nutrients

**Fig. 3:**
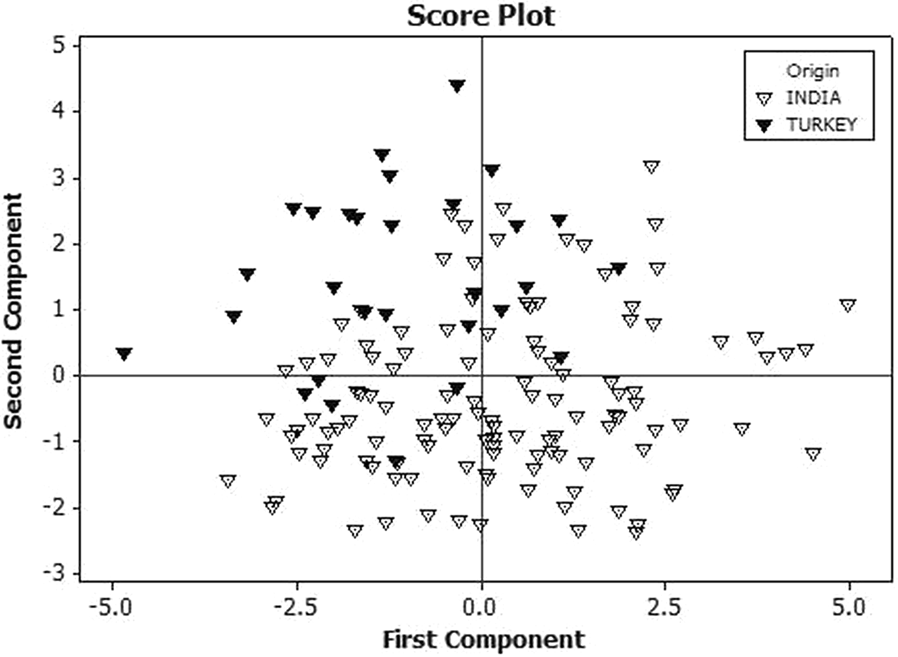
Scatterplot of first versus second principal component showing grouping of 121 Indian and 29 Turkish bread wheat genotypes

Principal component analysis was used to visualize the dispersion of genotypes based on origin (Fig. 3). The two dimensional PCA score plot, derived from multi-elemental and protein data revealed two main groups of Indian and Turkish genotypes. However, most of the Turkish varieties were tendentiously discriminated to negative scores concerning the first component, and positive scores for PC2. On the other hand, Indian samples were discriminated for negative scores for PC2, and positive scores concerning PC1. Separation of the involved wheat genotypes into two groups attributed to parental pedigree of the genotypes and differences in the growth conditions. However, association of some of the Indian and Turkish genotypes in close proximity to each other can be explained by the direct or indirect involvement of these varieties in worldwide modern breeding programs focusing elevated nutrient content. Also, Turkey being a centre of wheat domestication might have contributed towards the maintenance of these nutrients in Turkish wild and cultivated wheat that was further spread to the entire world.

**Fig. 4:**
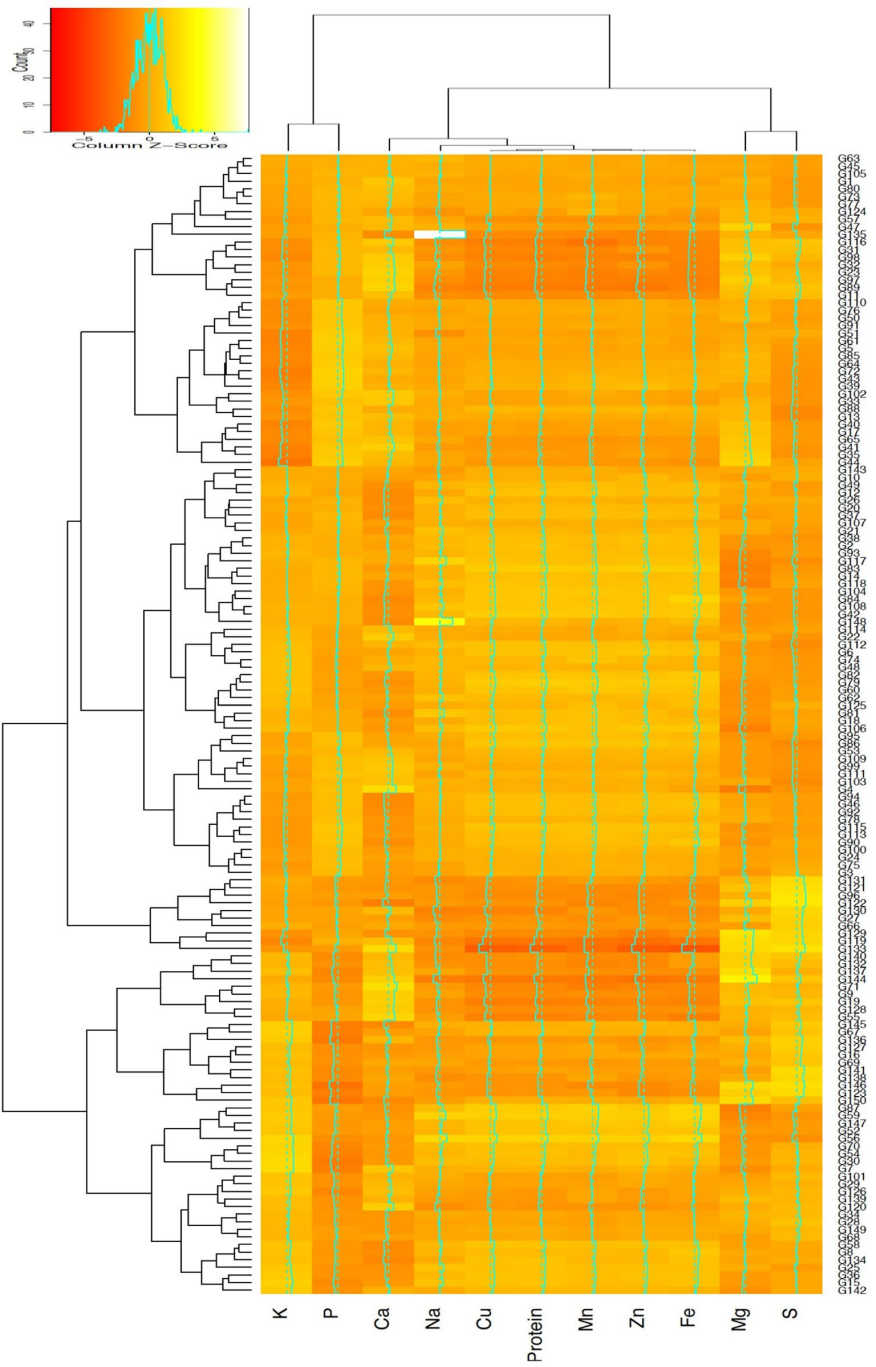
Heat-map and two-dimensional dendrogram of 121 Indian and 29 Turkish bread wheat genotypes for grain elemental and protein content. Dendrograms illustrate the relation between genotypes (*rows*) and nutrients (*columns*) using different *color shades* based on the average z-scores

Elemental and protein content summary of Indian and Turkish wheat varieties is demonstrated with the help of heat-map and two dimensional dendrogram (Fig. 4). Clustering of determined nutrients in wheat grains developed two main clusters. First cluster consisted of macronutrients, P and K, Second cluster was composed of three sub clusters. First, sub-cluster containing Mn, Zn and Fe and second sub-cluster containing Cu and protein was in close association with Ca and Na in comparison with the third cluster containing Mg and S. This grouping was strongly in accordance with the values obtained in Pearson Correlation analysis and P–K, Mn–Zn, Mg–S and Cu–protein were strongly correlated in both the analysis.

In hierarchical grouping, all the genotypes were divided into two main clusters, containing Indian and Turkish genotypes in line with the PCA. According to PCA, some of the Turkish genotypes were grouped with Indian cluster while few Indian varieties clustered with Turkish ones. Different subgroups were obtained in heatmap dendrogram that were similar to small clusters obtained in loading plot. Most of the genotypes showed average Z-scores (light orange color) for different elements. While some showed above average (yellowish color) Z-scores, others showed below average (reddish orange colors). However, Bayraktar showed extreme Z-score value for sodium content, none of the other genotypes showed extreme values or intensive colors. More values that are dissimilar were observed in two subgroups containing Turkish genotypes.

## Discussion

A number of authors have emphasized on the neglect of wheat nutritive value in successful breeding programs relying on yield increment (Welch and Graham 1999; Cakmak 2008; Cakmak et al. 2010). Although several authors contributed towards the determination of elemental content in bread wheat grains (Dikeman et al. 1982; Peterson et al. 1983; Monasterio and Graham 2000; Šramková et al. 2009), still more studies are required to illustrate variations in the elemental content and nutritive importance of wheat grains (Stallknecht et al. 1996). Focusing the issue, results of present study showed a considerable variation in elemental and protein content of wheat genotypes characterizing Indian and Turkish origin.

Series of macroelement and microelement concentrations of our bread wheat genotypes were in line with range obtained in several previous studies (Zhao et al. 2011). Most of the Turkish genotypes used in our study were similar to those involved by Harmankaya et al. (2012) and revealed similar elemental content. In addition, one of the varieties used in our study, Gerek_79 was included in previous grain nutrients analysis conducted by Chatzav et al. (2010). Although, there were slight differences among the values, but all the nutrient and protein contents could be compared. Similar range of nutrient concentrations of same genotypes in different experiments/studies directs towards varietal impact of wheat cultivars. Several fold variations among genotypes can be attributed to their genetic origins along with the harvest timings and agricultural practices.

Several scientists have emphasized on increasing the grain iron and zinc content up to 60.0 and 40.0 mg/kg, for positively affecting human health (Cakmak 2008). A number of genotypes involved in our study, namely DBW_77 (50.4 mg/kg), HW_4060 (59.4 mg/kg), HW_5202 (47.8 mg/kg), K_68 (44.0 mg/kg), K_8434 (41.3 mg/kg), K_9533 (40.0 mg/kg), MACS_6222 (45.8 mg/kg), 30KR8 (43.9 mg/kg), PBW_550 (43.3 mg/kg), UP_2511 (42.9 mg/kg) and Atilla (40.0 mg/kg) were found efficient reaching proposed Zn concentration. Although, none of the genotypes achieved projected Fe concentration, some were found closer to it like CBW_38 (47.1 mg/kg), HUW_37 (45.3 mg/kg), K_616 (49.1 mg/kg) and Ahmetaga (49.7 mg/kg). Only one Indian variety, UP_2696 was competent in both Fe (46.9 mg/kg) and Zn (40.9 mg/kg) concentrations, simultaneously. Such wheat sources with enhanced nutrients may be used directly as cultivated forms or indirectly as input material for breeding new, more nutritionally rich varieties of common wheat. Moreover, on the basis of recommended daily allowance (Table 3), it can be easily observed that several wheat genotypes can be consumed in the form of whole grain supply to fulfil a major part of their daily mineral requirements, mostly for microelements. Mineral profile of some of the experimental genotypes, like Atilla, AT052K2, HUW_55, K_8434, Murat, UP_2511, UP_2696 that are rich in three of the four studied micronutrients (Zn, Fe, Cu, Mn) can persuade people for whole grain consumption of wheat. Moreover, other genotypes with extremely high content of individual minerals like HW_4060 (59.4 mg/kg Zn), AT_052_K2 (15.6 mg/kg Cu and 65.6 mg/kg Mn), Ahmetaga (49.7 mg/kg Fe) can be considered by the people suffering from a particular nutrient deficiency and reach their recommended daily intake.

**Table 3:** Recommended daily allowance (RDA)

Element	RDA	Reference
Calcium	1000 mg/day	FAO/WHO
Potassium	3000–4000 mg/day	deMan (1999)
Phosphorus	700 mg/day	deMan (1999)
Magnesium	190–124 mg/day	FAO/WHO
Sodium	1500 mg/day	deMan (1999)
Manganese	2.3 mg/day	deMan (1999)
Copper	1.2 mg/day	deMan (1999)
Iron	1–3 mg/day	FAO/WHO
Zinc	36–150 µg/day	FAO/WHO

Several genes and physiological processes including nutrient uptake, their mobilisation, absorption in plant system and finally, storage in seeds highly contribute towards the correlation between grain nutrients and protein content (Bouis and Welch 2010). However, Cakmak et al. (2004) have focused on different genetic factors controlling nutrient efficiency and their accumulation in grains, their presence in different organs of plants may be affecting the entire mechanism of nutritional enhancement.

Positive correlation between Fe and Zn concentrations and strong association of grain iron and protein content of wheat grains in our experiment was concurrent with some previous studies performed on bread wheat (Morgounov et al. 2007; Zhao et al. 2009). This pointed towards the possibility of simultaneous improvement of both the nutrients. These associations can be ascribed to co-segregating alleles for different grain nutrients and co-localization of quantitative trait loci (QTLs). Several QTLs controlling Fe, Z and protein alliance are found in emmer wheat, double haploid populations and diploid wheat (Uauy et al. 2006; Morgounov et al. 2007; Peleg et al. 2009; Velu et al. 2014), however QTL information on grain Zn, iron and protein content in bread wheat is limited (Shi et al. 2008). In our study, contrary to previous experiments, wheat grain zinc and protein content were not found to be strongly correlated. Though, there were numerous genotypes like HW_5202, MACS_6222, 30KR8, UP_2696, Atilla with high zinc and protein content and showing positive correlation between the two, but total correlation estimated for all the genotypes was not strongly correlated. As zinc and protein content in wheat genotypes are considered to segregate together (Uauy et al. 2006; Peleg et al. 2009), this non-association may be linked to origin and may need further confirmation.

Not only, Fe and Zn with each other, but grain Cu and Mn also showed strong positive correlation with them. This can be linked to a major QTL on chromosome 5 controlling high Fe, Zn, Cu and Mn content in *T. monococcum* genotypes (Ozkan et al. 2007).

Positive correlation of sulphur with Fe and Zn content is of great significance due to the crucial role of sulphur containing aminoacids as promoters enhancing the Zn bioavailability (Welch and Graham 2004). This association is also crucial due to the involvement of S based aminoacid methionine in phytosiderophores production in cereals facilitating Fe and Zn mobilization in soil as well as in plants (Cakmak et al. 2010). Additionally, it has been illustrated that even marginal increase in sulphur supply leads to increased grain Zn and Fe concentrations (McDonald and Mousavvi Nik 2009). Positive association among Mg, P and Mn in our study was in accordance with the results obtained in the study conducted by Morgounov et al. (2007) on common wheat.

It is worth noting that macronutrient concentration in wheat is crucial for micronutrient balance, transport and bioavailability and their correlation should be considered. In our study, grain zinc was positively correlated with phosphorus. Co-localization of QTLs for grain Zn content with the QTLs for P content can be responsible for directing the positive association and will assist maintaining the wheat grain Zn and Phosphorus content simultaneously (Shi et al. 2008). Due to the accumulation of a major part of total P in the form of phytic acid, consequently phytate, Zn bioavailability is supposed to be affected (Stangoulis et al. 2007). Accumulation of grain Zn in aleurone layer and embryo, where phytate resides seems to contribute towards strong correlation between wheat grain Zn and Phosphorus content. These types of genetic, physical and physiological processes should be taken into account during nutrient enhancement targeted breeding programs.

## Conclusions

Simultaneous elemental and protein composition of Indian and Turkish wheat genotypes has been studied for the first time. As per the results obtained, nutritional profile distinguished the genotypes from the two countries. However, close association of some of the varieties justified the success of the global wheat exchange programs. Most of the associations between different nutrients were in accordance with the previously conducted nutritional analyses and in favour of the systematic interactions with in the plant system. However, non-correlation between Zn concentration and grain protein content pointed towards its regulation by some other genetic factors or processes. It was a crucial correlation required to be understood. Although several QTLs controlling grain nutrient content in diploid and tetraploid wheat have been identified, information on QTLs associated with high nutrient concentration in bread wheat is still limited. We suggest some wheat varieties with promising nutrient concentrations for future biofortification programs. Combined associations of several elements open the doors for their simultaneous improvement in breeding systems, however, individual concentration and effect of each component should be considered separately. Outcomes of present study will be supportive to plant breeders, food-processing industries and people consuming whole grain wheat products. Wheat classification based on their geographic origin employing elemental and protein analyses along is a competent approach that may indirectly support the target of removing malnutrition from the world in coming future.

## Methods

### Materials

A total of 150 hexaploid wheat genotypes including 121 Indian and 29 Turkish accessions were analyzed in this study for grain nutrients and protein content (Additional file 1: Table S1). Indian wheat varieties were obtained from Sam Higginbottom Institute of Agriculture, Technology and Sciences, Allahabad, India that were originally collected from different parts of the Northern wheat growing region of India rich in fertile alluvial soils. Turkish wheat varieties involved in the experiment were obtained from a wheat collection at Selcuk University, Konya, Turkey. These collected samples were directly used for further analysis.

Closed microwave system (Model: MARS-CEM Xpress) (CEM Corp., USA, 3100 Smith Farm Road, Matthews, NC) and Inductively Coupled Plasma-Atomic Emission Spectrometer (ICP-AES) (Vista-Pro Axial; Varian Pty Ltd, Australia) were used for elemental analysis.

### Determination of elemental and protein content

Collected samples of 150 bread wheat genotypes were washed separately using double distilled water and kept for drying in incubator at 60 °C for 1 day. For elemental analysis, dried seed samples were ground separately and fine powder samples were collected. Grinder was properly cleaned before grinding every sample. Teflon digestion vessels used for digestion were rinsed with 5 mL of concentrated HNO_3_. Approximately 0.1–0.2 g (weighed and recorded for each sample) of dried powder samples of whole wheat grains along with 5.0 mL of 65% nitric acid and 2.0 mL of 35% hydrogen peroxide were added to Teflon digestion vessel and kept for wet digestion in closed microwave system for about 30 min at 1800 W and 200 °C. Volumes of digested samples were adjusted to 20.0 mL using double distilled water. Three technical replicates of element concentrations of diluted samples were measured by ICP-AES with specific working conditions (Additional file 3: Table S3) and the concentrations were determined in parts per million (mg/kg) of dry matter. ICP-AES values were calibrated using 8346 durum wheat flour, 1567a wheat flour as the certified reference material, obtained from National Institute of Standards and Technology (Gaithersburg, MD, USA). Protein content of wheat grains was estimated using a LECO Tru-Spec CN analyzer (Leco Corp., St. Joseph, MI, USA). Instrument relies on the principle of dry combustion type utilizing thermal conductivity for nitrogen.

### Statistical analyses

All the data were subjected to statistical analysis to construct the elemental and protein profile to discriminate wheat origins and correlate between variables. Minitab 14 software was used to conduct major part of statistical analysis for comparison of elemental and protein content. All the nutrient and protein variables were examined for normal frequency distribution. Pearson correlation analysis was used to determine the correlations among different nutrients using selected wheat grains. The significance of these correlations was determined by ‘p’ value emphasizing the potency of linear association of two variables. Correlations are significant if p value is lower than 5%, however its significance increases with the decrease in the percentage. PCA was used to determine the associations among the grain protein and mineral nutrient concentrations. PCA is dependent on a correlation matrix and considered capable of revealing the underlying features of the variables known as principal components. Based on Eigen values, three main principal components were extracted explaining a major part of total variance and ensuring significant implementation of the data by each factor. Loading plots of elements and score plots of genotypes were drawn using multivariate system of PCA in Minitab 14 software. In addition, range, mean, median, skewness and kurtosis of grain element and protein concentrations in the 150 bread wheat genotypes was measured using descriptive statistics program of Minitab-14. Heatmap and cluster analysis was executed for visual separation of wheat genotypes employing function heatmap.2 and hclust in R software package version 2.15.1, respectively. This hierarchical clustering was based on the association of Euclidean distances of different genotypes in rows and several elements and protein content in columns. This study based on the element and protein analysis of 121 Indian and 29 Turkish bread wheat varieties would be helpful for wheat breeders and nutritionists to utilize in the biofortification programs. Scientists and food industries can effectively utilize the varietal disparities and nutrient correlations obtained in this study to conduct further research for wheat nutrients development.

## Additional files

**Additional file 1.** **Table S1**. Element and protein content of 121 Indian and 29 Turkish bread wheat genotypes estimated by ICP-AES and LECO analyser, respectively.

**Additional file 2.** **Table S2**. Loadings of the elemental and protein variables of the first three Principal Components (PC) for 121 Indian and 29 Turkish bread wheat genotypes.

**Additional file 3.** **Table S3**. Working conditions and specifications of ICP-AES in this experiment.
