# Supplementary material for: Assessment of genetic variability for grain nutrients from diverse regions: potential for wheat improvement
Source: Springerplus. 2016 Nov 3;5(1):1912. doi: 10.1186/s40064-016-3586-2 (PMC5095102; doi:10.1186/s40064-016-3586-2)
Supplement: Supplementary file 1 — Additional file 1. Table S1. Element and protein content of 121 Indian and 29 Turkish bread wheat genotypes estimated by ICP-AES and LECO analyser, respectively. [file 40064_2016_3586_MOESM1_ESM.docx]

**TableS1.** Element and protein content of 121 Indian and 29 Turkish bread wheat genotypes estimated by ICP-AES and LECO analyser, respectively.

| **Codes** | **Cultivar** | **Origin** | **Ca** | | **K** | | **Mg** | | **Na** | | **P** | | **S** | | **Zn** | | **Cu** | | **Fe** | | **Mn** | | **GPC** | |
| --- | --- | --- | --- | --- | --- | --- | --- | --- | --- | --- | --- | --- | --- | --- | --- | --- | --- | --- | --- | --- | --- | --- | --- | --- |
|  |  |  | **Mean** | | **Mean** | | **Mean** | | **Mean** | | **Mean** | | **Mean** | | **Mean** | | **Mean** | | **Mean** | | **Mean** | | **Mean** | |
|  |  |  | **SD** | | **SD** | | **SD** | | **SD** | | **SD** | | **SD** | | **SD** | | **SD** | | **SD** | | **SD** | | **SD** | |
| **G1** | **AAI_12** | INDIA | 478.0 | 3.7 | 3951.5 | 52.8 | 1848.7 | 12.9 | 154.0 | 7.7 | 3466.4 | 18.8 | 1115.5 | 5.4 | 25.4 | 0.2 | 4.7 | 0.1 | 22.4 | 0.2 | 43.1 | 0.4 | 10.6 | 0.3 |
| **G2** | **AAI_16** | INDIA | 264.2 | 0.5 | 4409.3 | 34.7 | 1766.1 | 18.1 | 163.7 | 2.5 | 3737.4 | 22.6 | 1179.8 | 8.7 | 24.6 | 0.1 | 4.2 | 0.0 | 23.0 | 0.1 | 24.3 | 0.1 | 13.3 | 0.2 |
| **G3** | **AAI_23** | INDIA | 238.7 | 1.7 | 3715.9 | 26.0 | 1634.1 | 1.9 | 121.5 | 3.7 | 3525.3 | 34.0 | 1311.9 | 6.3 | 26.1 | 0.2 | 6.4 | 0.1 | 25.8 | 0.0 | 36.7 | 0.2 | 12.1 | 0.3 |
| **G4** | **AAI_28** | INDIA | 663.5 | 1.9 | 4462.5 | 6.3 | 1723.5 | 0.9 | 171.1 | 1.0 | 4265.3 | 47.1 | 1221.2 | 7.4 | 33.3 | 0.4 | 5.7 | 0.1 | 9.3 | 0.0 | 36.8 | 0.1 | 11.4 | 0.2 |
| **G5** | **AAI_347** | INDIA | 478.1 | 1.9 | 3733.5 | 3.4 | 1957.5 | 1.6 | 165.5 | 2.5 | 4075.8 | 50.5 | 1202.6 | 16.0 | 33.0 | 0.1 | 5.6 | 0.1 | 29.4 | 0.3 | 36.8 | 0.2 | 13.5 | 0.4 |
| **G6** | **C_306** | INDIA | 236.3 | 1.4 | 3943.8 | 46.9 | 1566.2 | 6.2 | 116.5 | 3.7 | 3090.5 | 18.0 | 966.5 | 12.0 | 32.2 | 0.1 | 4.8 | 0.1 | 21.9 | 0.2 | 34.7 | 0.3 | 10.7 | 0.4 |
| **G7** | **CBW_24** | INDIA | 430.4 | 1.3 | 4203.8 | 8.7 | 1514.7 | 13.8 | 139.2 | 1.7 | 2623.1 | 16.7 | 1227.0 | 7.8 | 31.5 | 0.1 | 6.4 | 0.0 | 18.5 | 0.2 | 25.8 | 0.1 | 11.3 | 0.2 |
| **G8** | **CBW_38** | INDIA | 137.3 | 2.3 | 3831.6 | 27.4 | 1624.8 | 19.3 | 133.6 | 4.3 | 2840.4 | 22.7 | 1119.4 | 6.1 | 21.4 | 0.1 | 6.0 | 0.1 | 47.1 | 0.2 | 32.6 | 0.1 | 11.1 | 0.4 |
| **G9** | **DBW_52** | INDIA | 524.4 | 1.7 | 3889.9 | 20.3 | 1860.0 | 10.7 | 177.8 | 1.4 | 2944.4 | 31.8 | 1365.0 | 6.1 | 31.0 | 0.4 | 5.8 | 0.1 | 26.3 | 0.1 | 39.0 | 0.3 | 9.5 | 0.3 |
| **G10** | **DBW_77** | INDIA | 305.4 | 2.1 | 4584.9 | 30.6 | 1975.4 | 8.1 | 148.0 | 5.8 | 3943.6 | 41.5 | 1516.8 | 6.2 | 50.4 | 0.2 | 7.5 | 0.1 | 26.6 | 0.3 | 36.5 | 0.2 | 14.9 | 0.2 |
| **G11** | **FLW_15** | INDIA | 466.3 | 1.1 | 3651.8 | 27.9 | 1884.8 | 11.9 | 140.0 | 1.7 | 3318.4 | 7.0 | 1460.0 | 12.0 | 43.9 | 0.2 | 7.5 | 0.1 | 37.3 | 0.1 | 40.4 | 0.0 | 15.8 | 0.7 |
| **G12** | **HD_1941** | INDIA | 114.6 | 1.1 | 3458 | 45.6 | 1501.9 | 6.4 | 91.2 | 5.6 | 2812.9 | 19.3 | 982.0 | 11.0 | 19.8 | 0.2 | 4.7 | 0.1 | 20.2 | 0.2 | 32.7 | 0.2 | 11.6 | 0.2 |
| **G13** | **HD_2285** | INDIA | 444.7 | 2.7 | 3781.9 | 9.0 | 1946.2 | 6.3 | 167.8 | 3.8 | 3827.4 | 19.4 | 994.7 | 4.8 | 32.5 | 0.2 | 8.2 | 0.0 | 39.2 | 0.3 | 40.3 | 0.3 | 10.4 | 0.3 |
| **G14** | **HD_2687** | INDIA | 284.2 | 1.8 | 4229.0 | 65.0 | 1627.6 | 18.2 | 139.9 | 8.7 | 3821.9 | 2.7 | 1208.6 | 13.0 | 26.2 | 0.3 | 4.7 | 0.1 | 28.3 | 0.4 | 37.0 | 0.5 | 10.0 | 0.6 |
| **G15** | **HD_2733** | INDIA | 194.3 | 1.9 | 4031.9 | 26.0 | 1535.9 | 12.6 | 153.6 | 3.4 | 2703.8 | 12.4 | 1120.4 | 3.8 | 21.6 | 0.1 | 5.7 | 0.1 | 27.0 | 0.1 | 25.2 | 0.1 | 13.3 | 0.2 |
| **G16** | **HD_2781** | INDIA | 229.2 | 1.6 | 3657.5 | 16.5 | 1575.2 | 12.4 | 123.5 | 2.3 | 2640.8 | 17.6 | 1371.3 | 8.7 | 33.4 | 0.1 | 7.5 | 0.0 | 41.4 | 0.3 | 42.9 | 0.2 | 13.0 | 0.3 |
| **G17** | **HD_2888** | INDIA | 398.4 | 1.9 | 3516.1 | 19.4 | 1932.1 | 15.3 | 141.4 | 2.0 | 3665.9 | 38.5 | 1122.3 | 12.0 | 33.5 | 0.2 | 5.9 | 0.1 | 32.8 | 0.1 | 35.1 | 0.3 | 9.8 | 0.4 |
| **G18** | **HD_2891** | INDIA | 198.1 | 0.8 | 3589.3 | 19.8 | 1406.6 | 10.3 | 111.2 | 2.3 | 3010.9 | 14.1 | 1071.6 | 7.2 | 26.3 | 0.2 | 5.4 | 0.0 | 22.2 | 0.2 | 42.7 | 0.3 | 12.5 | 0.2 |
| **G19** | **HD_2932** | INDIA | 491.1 | 3.1 | 3379.4 | 25.2 | 1693.2 | 5.1 | 149.9 | 3.6 | 2645.3 | 26.1 | 1340.1 | 5.3 | 34.2 | 0.2 | 6.1 | 0.1 | 35.0 | 0.3 | 36.2 | 0.3 | 11.7 | 0.3 |
| **G20** | **HD_2967** | INDIA | 151 | 0.5 | 3579.6 | 27.3 | 1558.8 | 3.5 | 123.4 | 3.5 | 3098.6 | 21.2 | 1084.3 | 13.0 | 19.6 | 0.1 | 4.6 | 0.1 | 23.8 | 0.2 | 33.9 | 0.3 | 9.8 | 0.3 |
| **G21** | **HD_2985** | INDIA | 191.9 | 1.5 | 3849.2 | 20.0 | 1757.0 | 12.7 | 173.7 | 3.1 | 3421.3 | 15.8 | 1101.6 | 5.9 | 23.5 | 0.2 | 5.3 | 0.0 | 30.8 | 0.0 | 24.3 | 0.1 | 11.7 | 0.3 |
| **G22** | **HI_1563** | INDIA | 434.2 | 1.5 | 3524.1 | 29.7 | 1545.7 | 15.5 | 141.0 | 2.8 | 2815.2 | 31.6 | 919.7 | 2.7 | 27.9 | 0.1 | 4.5 | 0.0 | 21.8 | 0.1 | 29.5 | 0.2 | 10.3 | 0.5 |
| **G23** | **HS_420** | INDIA | 567.0 | 1.9 | 3514.9 | 11.7 | 1929.0 | 3.9 | 167.5 | 1.0 | 3404.9 | 29.5 | 1367.6 | 17.0 | 34.8 | 0.1 | 5.2 | 0.0 | 31.0 | 0.1 | 32.1 | 0.1 | 14.4 | 0.4 |
| **G24** | **HUW_213** | INDIA | 278.1 | 1.0 | 3818.1 | 7.7 | 1721.2 | 8.6 | 151.5 | 1.0 | 3620.4 | 36.8 | 1231.8 | 9.9 | 24.0 | 0.2 | 5.4 | 0.0 | 25.0 | 0.2 | 42.8 | 0.0 | 11.8 | 0.2 |
| **G25** | **HUW_234** | INDIA | 142.3 | 1.1 | 3692.7 | 24.9 | 1567.1 | 4.9 | 147.8 | 1.1 | 2651.9 | 12.9 | 965.6 | 1.9 | 22.8 | 0.1 | 3.9 | 0.0 | 23.7 | 0.2 | 26.7 | 0.2 | 12.8 | 0.2 |
| **G26** | **HUW_510** | INDIA | 130.2 | 0.4 | 3104.9 | 25.3 | 1445.1 | 2.4 | 150.7 | 3.2 | 2794.2 | 9.7 | 1028.7 | 9.3 | 20.1 | 0.1 | 5.6 | 0.1 | 22.3 | 0.1 | 32.2 | 0.1 | 12.2 | 0.3 |
| **G27** | **HW_2006** | INDIA | 260.7 | 2.5 | 3216.9 | 22.0 | 1633.9 | 6.8 | 106.2 | 2.1 | 2726.3 | 4.8 | 1403.4 | 12.0 | 33.7 | 0.2 | 6.1 | 0.1 | 27.5 | 0.1 | 29.5 | 0.1 | 13.7 | 0.3 |
| **G28** | **HW_2045** | INDIA | 213.9 | 1.6 | 3487.0 | 6.0 | 1568.0 | 5.2 | 126.9 | 2.9 | 2633.5 | 20.5 | 1298.4 | 16.0 | 34.4 | 0.3 | 9.2 | 0.0 | 33.7 | 0.2 | 31.5 | 0.3 | 13.3 | 0.5 |
| **G29** | **HW_2071** | INDIA | 393.4 | 3.1 | 3949.1 | 19.5 | 1681.4 | 7.0 | 150.8 | 1.8 | 2869.9 | 11.8 | 1260.4 | 11.0 | 36.3 | 0.4 | 6.3 | 0.0 | 29.9 | 0.2 | 33.5 | 0.2 | 12.8 | 0.2 |
| **G30** | **HW_4024** | INDIA | 172.8 | 1.5 | 3660.2 | 19.7 | 1386.1 | 2.7 | 132.0 | 1.5 | 2227.2 | 15.4 | 1127.0 | 14.0 | 33.6 | 0.1 | 4.9 | 0.0 | 19.6 | 0.1 | 29.5 | 0.1 | 13.0 | 0.5 |
| **G31** | **HW_4060** | INDIA | 489.0 | 3.9 | 3848.4 | 20.9 | 2110.1 | 15.9 | 181.8 | 2.5 | 3802.5 | 27.2 | 1630.3 | 23.0 | 59.4 | 0.6 | 7.8 | 0.1 | 35.2 | 0.2 | 41.2 | 0.4 | 12.7 | 0.3 |
| **G32** | **HW_5202** | INDIA | 587.5 | 3.7 | 3909.0 | 36.7 | 2073.2 | 7.2 | 199.0 | 6.1 | 3760.5 | 23.7 | 1411.3 | 17.0 | 47.8 | 0.3 | 6.1 | 0.0 | 24.8 | 0.2 | 36.3 | 0.1 | 14.9 | 0.4 |
| **G33** | **K_0307** | INDIA | 502.1 | 2.5 | 3920.6 | 38.1 | 1958.1 | 12.8 | 164.2 | 4.4 | 3859.6 | 27.3 | 1110.9 | 5.5 | 29.5 | 0.3 | 4.0 | 0.1 | 24.0 | 0.2 | 41.1 | 0.2 | 9.8 | 0.3 |
| **G34** | **K_0402** | INDIA | 199.6 | 0.6 | 3371.6 | 13.3 | 1551.9 | 2.4 | 131.2 | 2.6 | 2562.8 | 5.4 | 1181.3 | 17.0 | 23.9 | 0.1 | 6.6 | 0.1 | 23.5 | 0.1 | 24.7 | 0.0 | 12.1 | 0.2 |
| **G35** | **K_0424** | INDIA | 411.1 | 2.1 | 3170.4 | 8.0 | 1876.5 | 4.4 | 150.4 | 3.5 | 3456.2 | 12.8 | 990.2 | 3.8 | 33.6 | 0.2 | 4.3 | 0.1 | 25.0 | 0.0 | 29.1 | 0.1 | 12.0 | 0.2 |
| **G36** | **K_0607** | INDIA | 160.8 | 1.8 | 4078.5 | 48.8 | 1593.0 | 8.7 | 131.3 | 5.3 | 2904.5 | 33.2 | 1166.1 | 12.0 | 23.8 | 0.0 | 7.0 | 0.1 | 24.1 | 0.2 | 27.5 | 0.2 | 10.0 | 0.2 |
| **G37** | **K_0906** | INDIA | 132.3 | 0.8 | 3800.8 | 22.0 | 1655.9 | 6.5 | 131.6 | 3.9 | 3481.3 | 35.0 | 1167.2 | 2.2 | 25.3 | 0.1 | 6.2 | 0.1 | 21.7 | 0.1 | 37.1 | 0.3 | 11.2 | 0.3 |
| **G38** | **K_65** | INDIA | 281.9 | 1.9 | 4288.9 | 31.2 | 1755.0 | 13.2 | 153.2 | 4.1 | 3626.7 | 13.4 | 1105.3 | 6.9 | 33.6 | 0.3 | 5.9 | 0.1 | 29.8 | 0.2 | 34.2 | 0.2 | 10.4 | 0.3 |
| **G39** | **K_68** | INDIA | 396.7 | 2.3 | 3997.5 | 32.4 | 1968.8 | 7.7 | 143.3 | 3.8 | 4341.7 | 42.9 | 1142 | 6.9 | 44.0 | 0.4 | 6.0 | 0.0 | 28.7 | 0.1 | 44.7 | 0.2 | 10.6 | 0.2 |
| **G40** | **K_7903** | INDIA | 369.8 | 1.3 | 3262.3 | 46.4 | 1838.4 | 10.0 | 135.8 | 5.2 | 3515.8 | 23.2 | 1038.3 | 11.0 | 27.9 | 0.2 | 5.9 | 0.1 | 40.3 | 0.3 | 38.4 | 0.2 | 8.7 | 0.4 |
| **G41** | **K_8027** | INDIA | 533.2 | 2.3 | 3402.8 | 17.1 | 1959.1 | 11.1 | 143.2 | 1.3 | 3670.9 | 45.9 | 1066.8 | 10.0 | 36.7 | 0.3 | 5.9 | 0.0 | 38.3 | 0.1 | 42.4 | 0.1 | 10.7 | 0.4 |
| **G42** | **K_816** | INDIA | 140.0 | 1.3 | 4237 | 34.1 | 1701.7 | 18.3 | 131.7 | 3.3 | 3622.2 | 37.7 | 1087.6 | 14.0 | 19.4 | 0.1 | 5.0 | 0.0 | 22.7 | 0.3 | 32.7 | 0.1 | 11.2 | 0.2 |
| **G43** | **K_8434** | INDIA | 394.0 | 1.8 | 4249.0 | 15.9 | 2251.1 | 13.0 | 154.1 | 1.2 | 4720.3 | 31.6 | 1290.5 | 17.0 | 41.3 | 0.3 | 6.6 | 0.0 | 39.2 | 0.1 | 58.6 | 0.2 | 11.4 | 0.2 |
| **G44** | **K_8962** | INDIA | 440.7 | 2.8 | 3495.1 | 8.9 | 2174.9 | 11.5 | 185.9 | 3.0 | 4016.9 | 14.0 | 1273.6 | 4.2 | 31.6 | 0.1 | 5.6 | 0.1 | 25.7 | 0.1 | 41.5 | 0.2 | 11.9 | 0.3 |
| **G45** | **K_9006** | INDIA | 353.5 | 1.2 | 3834.5 | 34.4 | 1755.1 | 7.8 | 154.9 | 2.7 | 3376.8 | 36.8 | 1139.0 | 14.0 | 25.4 | 0.1 | 5.6 | 0.1 | 25.6 | 0.1 | 28.5 | 0.1 | 12.0 | 0.4 |
| **G46** | **K_9107** | INDIA | 148.7 | 1.4 | 4075.8 | 30.7 | 1875.3 | 1.6 | 137.3 | 3.2 | 3930.5 | 42.2 | 1198.2 | 15.0 | 29.7 | 0.0 | 6.7 | 0.0 | 37.2 | 0.2 | 29.9 | 0.1 | 13.3 | 0.5 |
| **G47** | **K_9351** | INDIA | 391.5 | 2.8 | 3596.1 | 26.1 | 1957.9 | 18.8 | 174.8 | 2.5 | 3227.8 | 9.3 | 1004.3 | 19.0 | 23.7 | 0.1 | 5.9 | 0.1 | 34.6 | 0.4 | 30.1 | 0.2 | 9.4 | 0.4 |
| **G48** | **K_9423** | INDIA | 332 | 2.9 | 3613.1 | 17.4 | 1469.7 | 7.5 | 141.9 | 2.1 | 2788.9 | 26.4 | 945.9 | 9.6 | 21.4 | 0.2 | 4.2 | 0.0 | 21.5 | 0.1 | 30.6 | 0.0 | 12.3 | 0.4 |
| **G49** | **K_9465** | INDIA | 146.7 | 0.1 | 3404.2 | 34.0 | 1537.8 | 12.8 | 125.2 | 3.7 | 2838.5 | 16.4 | 940.4 | 5.0 | 19.9 | 0.2 | 4.7 | 0.0 | 25.1 | 0.1 | 36.8 | 0.3 | 11.0 | 0.3 |
| **G50** | **K_9533** | INDIA | 384.3 | 1.2 | 3926.4 | 30.8 | 2063.2 | 11.2 | 167.8 | 2.2 | 4064.0 | 21.7 | 1246.0 | 0.3 | 40.0 | 0.1 | 5.4 | 0.0 | 28.2 | 0.2 | 42.3 | 0.3 | 13.3 | 0.2 |
| **G51** | **CLN_5** | INDIA | 405.7 | 3.8 | 3634.1 | 11.3 | 1889.9 | 8.8 | 110.1 | 2.4 | 3993.7 | 37.6 | 1365.3 | 7.2 | 37.2 | 0.3 | 5.9 | 0.1 | 36.8 | 0.2 | 33.7 | 0.3 | 10.8 | 0.3 |
| **G52** | **KRL_210** | INDIA | 196.9 | 1.0 | 3708.4 | 25.3 | 1463.1 | 10.1 | 114.9 | 3.7 | 2748.0 | 26.8 | 954.5 | 5.4 | 23.1 | 0.1 | 4.8 | 0.1 | 24.6 | 0.1 | 31.0 | 0.4 | 9.8 | 0.3 |
| **G53** | **KRL_213** | INDIA | 393.7 | 0.7 | 4051.8 | 7.5 | 1725.6 | 12.6 | 134.4 | 2.0 | 3605.6 | 31.3 | 1044.4 | 22.0 | 28.6 | 0.4 | 5.3 | 0.1 | 36.4 | 0.1 | 37.2 | 0.1 | 13.5 | 0.3 |
| **G54** | **LOK_45** | INDIA | 192.6 | 2.1 | 4295.3 | 63.1 | 1591.0 | 14.8 | 136.7 | 8.1 | 2736.6 | 25.1 | 1321.2 | 10.0 | 33.5 | 0.3 | 5.4 | 0.1 | 30.7 | 0.4 | 25.1 | 0.3 | 15.2 | 0.2 |
| **G55** | **MACS_6222** | INDIA | 635.1 | 2.3 | 4099 | 6.9 | 2000.6 | 8.8 | 200.7 | 1.0 | 3489.9 | 24.9 | 1569.5 | 18.0 | 45.8 | 0.1 | 5.5 | 0.1 | 31.4 | 0.1 | 46.0 | 0.3 | 13.2 | 0.3 |
| **G56** | **NW_1014** | INDIA | 152.0 | 0.5 | 4168.5 | 39.4 | 1491.3 | 3.1 | 175.4 | 5.1 | 2789.7 | 12.6 | 901.0 | 13.0 | 19.0 | 0.1 | 5.6 | 0.0 | 33.9 | 0.2 | 31.4 | 0.2 | 11.8 | 0.2 |
| **G57** | **PBW_343** | INDIA | 490.7 | 2.9 | 3982.2 | 35.0 | 2035.0 | 20.7 | 198.4 | 3.2 | 3788.9 | 45.8 | 1469.8 | 2.3 | 34.3 | 0.1 | 6.7 | 0.1 | 39.2 | 0.2 | 38.9 | 0.2 | 12.3 | 0.1 |
| **G58** | **PBW_502** | INDIA | 112.4 | 1.1 | 3876.8 | 24.4 | 1566.9 | 16.3 | 130.2 | 2.6 | 2831.1 | 3.4 | 1131.9 | 2.5 | 21.5 | 0.1 | 7.9 | 0.1 | 22.2 | 0.1 | 29.6 | 0.1 | 12.5 | 0.5 |
| **G59** | **PBW_524** | INDIA | 155.3 | 1.3 | 4017.3 | 21.2 | 1412.3 | 5.8 | 164.8 | 3.9 | 2914.8 | 9.8 | 931.9 | 7.8 | 20.2 | 0.2 | 6.2 | 0.0 | 33.1 | 0.1 | 32.1 | 0.2 | 10.4 | 0.5 |
| **G60** | **WR_1451** | INDIA | 192.3 | 0.9 | 3899.9 | 10.1 | 1483.0 | 4.0 | 109.1 | 1.9 | 3008.2 | 7.7 | 1074.6 | 6.5 | 26.4 | 0.1 | 5.1 | 0.0 | 23.8 | 0.2 | 41.9 | 0.3 | 9.6 | 0.3 |
| **G61** | **30KR8** | INDIA | 464.4 | 3.5 | 4045.6 | 5.6 | 2079.1 | 13.2 | 167.2 | 1.5 | 4481.4 | 30.7 | 1354.3 | 5.4 | 44 | 0.2 | 5.3 | 0.1 | 22.1 | 0.2 | 38.6 | 0.2 | 14.4 | 0.5 |
| **G62** | **AAI2** | INDIA | 244.4 | 7.6 | 3934.1 | 19.5 | 1541.5 | 7.3 | 171.9 | 5.1 | 3168.9 | 55.6 | 1102.0 | 12.0 | 30.5 | 0.5 | 8.1 | 0.1 | 36.6 | 0.9 | 34.7 | 0.7 | 12.6 | 0.3 |
| **G63** | **AKAW4006** | INDIA | 370.1 | 2.3 | 3871.0 | 23.0 | 1726.1 | 3.0 | 176.8 | 1.4 | 3381.4 | 25.8 | 1142.9 | 26.0 | 24.3 | 0.3 | 3.8 | 0.0 | 20.2 | 0.1 | 33.8 | 0.1 | 12.0 | 0.3 |
| **G64** | **DBW14** | INDIA | 369.0 | 2.5 | 3778.8 | 32.7 | 1994.2 | 12.7 | 136.6 | 1.7 | 4100.5 | 37.5 | 1178.4 | 15.0 | 33.4 | 0.4 | 6.1 | 0.0 | 37.6 | 0.3 | 34.0 | 0.3 | 10.5 | 0.2 |
| **G65** | **DBW39** | INDIA | 486.6 | 4.2 | 3627.4 | 50.8 | 2021.3 | 18.6 | 182.1 | 6.6 | 3693.3 | 40.1 | 1164.6 | 11.0 | 33.3 | 0.3 | 6.1 | 0.1 | 41.5 | 0.5 | 33.4 | 0.4 | 11.1 | 0.5 |
| **G66** | **DDK1025** | INDIA | 350.7 | 1.4 | 3976.8 | 2.8 | 1729.0 | 6.7 | 148.8 | 1.7 | 3226.7 | 12.2 | 1642.5 | 13.0 | 27.9 | 0.1 | 6.0 | 0.1 | 38.0 | 0.1 | 33.4 | 0.1 | 14.2 | 0.3 |
| **G67** | **GW03_12** | INDIA | 211.5 | 1.6 | 3749.3 | 11.8 | 1510.3 | 3.6 | 102.2 | 2.0 | 2418.4 | 20.3 | 1297.4 | 16.0 | 18.5 | 0.2 | 5.1 | 0.0 | 22.1 | 0.1 | 25 | 0.2 | 10.7 | 0.3 |
| **G68** | **GW03_2** | INDIA | 239.4 | 1.3 | 3424.5 | 40.0 | 1459.0 | 17.9 | 124.0 | 6.5 | 2721.5 | 28.0 | 1208.9 | 12.0 | 25.7 | 0.2 | 6.1 | 0.1 | 27.9 | 0.2 | 26.4 | 0.2 | 12.7 | 0.3 |
| **G69** | **GW03_3** | INDIA | 308.5 | 3.2 | 3695.3 | 31.8 | 1673.0 | 4.9 | 149.4 | 5.4 | 2727.2 | 26.1 | 1400.3 | 4.5 | 24.2 | 0.1 | 5.5 | 0.1 | 27.4 | 0.3 | 30.8 | 0.1 | 11.9 | 0.2 |
| **G70** | **GW03_4** | INDIA | 200.8 | 2.4 | 4069.2 | 4.8 | 1539.9 | 9.8 | 142.5 | 1.7 | 2654.9 | 5.3 | 1200.2 | 10.0 | 22.9 | 0.1 | 7.2 | 0.1 | 24.7 | 0.2 | 32.3 | 0.1 | 11.2 | 0.3 |
| **G71** | **GW03_9** | INDIA | 637.1 | 3.7 | 3944.1 | 39.7 | 1895.1 | 10.8 | 172.0 | 2.0 | 3070.5 | 14.5 | 1365.3 | 8.9 | 24.5 | 0.1 | 6.3 | 0.1 | 36.3 | 0.3 | 22.5 | 0.1 | 15.0 | 0.3 |
| **G72** | **HD2177** | INDIA | 390.2 | 1.1 | 3586.5 | 12.3 | 1963.8 | 15.6 | 143.5 | 0.6 | 4068.3 | 24.7 | 1083.2 | 8.0 | 32.8 | 0.3 | 6.1 | 0.0 | 29.6 | 0.2 | 41.8 | 0.2 | 10.4 | 0.3 |
| **G73** | **HD2236** | INDIA | 400.1 | 2.4 | 3748.2 | 31.2 | 1727.8 | 18.7 | 132.9 | 5.4 | 3493.8 | 10.2 | 1086.3 | 3.1 | 25.8 | 0.3 | 4.1 | 0.1 | 20.2 | 0.0 | 55.0 | 0.3 | 9.3 | 0.3 |
| **G74** | **HD2270** | INDIA | 259.9 | 0.6 | 3404.2 | 11.6 | 1389.3 | 10.4 | 128.8 | 3.5 | 2624.5 | 19.1 | 871.6 | 2.1 | 22.0 | 0.2 | 3.3 | 0.0 | 18.2 | 0.1 | 47.8 | 0.1 | 9.6 | 0.2 |
| **G75** | **HD2307** | INDIA | 232.2 | 3.1 | 3551.1 | 40.9 | 1650.3 | 3.2 | 120.3 | 6.3 | 3477.5 | 52.3 | 1222.6 | 14.0 | 28.3 | 0.1 | 5.5 | 0.1 | 26.2 | 0.3 | 42.7 | 0.3 | 10.4 | 0.3 |
| **G76** | **HD2329** | INDIA | 308.2 | 1.4 | 3663.0 | 9.5 | 1868.5 | 7.7 | 131.1 | 1.9 | 3773.6 | 46.4 | 1199.9 | 5.0 | 30.0 | 0.2 | 6.7 | 0.0 | 31.6 | 0.1 | 43.4 | 0.2 | 11.1 | 0.2 |
| **G77** | **HD2380** | INDIA | 343.8 | 1.3 | 3481.1 | 17.1 | 1680.9 | 1.9 | 128.8 | 2.7 | 3215.0 | 10.6 | 1027.6 | 6.7 | 22.0 | 0.1 | 3.3 | 0.0 | 28.6 | 0.1 | 38.9 | 0.2 | 10.6 | 0.3 |
| **G78** | **HD2402** | INDIA | 204.9 | 1.4 | 3664.1 | 73.4 | 1787.7 | 5.4 | 136.2 | 9.6 | 3595.4 | 18.8 | 1165.3 | 16.0 | 30.1 | 0.4 | 5.8 | 0.2 | 32.1 | 0.3 | 41.1 | 0.4 | 9.6 | 0.3 |
| **G79** | **HD2501** | INDIA | 115.7 | 0.5 | 3667.7 | 16.1 | 1420.1 | 5.4 | 100.2 | 0.7 | 2867.8 | 17.4 | 953.4 | 9.9 | 16.7 | 0.3 | 4.2 | 0.0 | 17.3 | 0.0 | 29.1 | 0.1 | 9.8 | 0.2 |
| **G80** | **HD2643** | INDIA | 390.7 | 3.7 | 3504.1 | 16.4 | 1639.5 | 14.6 | 151.1 | 4.2 | 3242.7 | 32.3 | 1033.3 | 4.6 | 24.6 | 0.0 | 5.0 | 0.0 | 34.8 | 0.2 | 33.0 | 0.4 | 9.0 | 0.4 |
| **G81** | **HD2881** | INDIA | 145.5 | 1.0 | 3789.3 | 13.5 | 1542.4 | 14.3 | 174.6 | 0.7 | 3188.6 | 31.2 | 1100.9 | 6.6 | 22.1 | 0.1 | 5.8 | 0.0 | 9.2 | 0.0 | 39.1 | 0.1 | 12.9 | 0.4 |
| **G82** | **HUW12** | INDIA | 169.2 | 0.6 | 3611.5 | 41.1 | 1441.2 | 4.6 | 112.2 | 4.7 | 2801.2 | 17.2 | 945.6 | 3.0 | 21.1 | 0.1 | 3.7 | 0.0 | 35.9 | 0.0 | 33.3 | 0.1 | 9.9 | 0.4 |
| **G83** | **HUW251** | INDIA | 208.9 | 1.0 | 4473.5 | 46.7 | 1668.1 | 1.2 | 128.6 | 3.9 | 4018.5 | 15.7 | 1196.2 | 12.0 | 32.8 | 0.2 | 6.4 | 0.1 | 31.8 | 0.2 | 35.3 | 0.2 | 14.8 | 0.3 |
| **G84** | **HUW37** | INDIA | 105.9 | 0.7 | 3747.9 | 59.9 | 1608.9 | 7.0 | 121.6 | 6.9 | 3236.3 | 31.7 | 971.1 | 3.1 | 22.9 | 0.2 | 5.4 | 0.1 | 45.3 | 0.2 | 27.0 | 0.2 | 10.2 | 0.5 |
| **G85** | **HUW468** | INDIA | 393.4 | 1.7 | 3379.8 | 40.7 | 1799.7 | 9.5 | 141.8 | 4.1 | 3611.5 | 33.6 | 1060.4 | 12.0 | 29.7 | 0.3 | 6.4 | 0.0 | 35.0 | 0.4 | 37.9 | 0.1 | 11.9 | 0.4 |
| **G86** | **HUW533** | INDIA | 231.7 | 1.8 | 3382.6 | 11.7 | 1454.9 | 13.8 | 85.3 | 3.5 | 3181.4 | 0.6 | 837.4 | 11.0 | 24.2 | 0.1 | 4.3 | 0.1 | 17.2 | 0.0 | 32.9 | 0.0 | 9.8 | 0.5 |
| **G87** | **HUW55** | INDIA | 137.8 | 0.4 | 4131.2 | 21.5 | 1429.5 | 11.6 | 136.1 | 3.6 | 3083.5 | 12.3 | 1110.0 | 7.6 | 18.9 | 0.1 | 7.7 | 0.0 | 39.7 | 0.1 | 44.3 | 0.2 | 11.1 | 0.2 |
| **G88** | **K01006** | INDIA | 353.0 | 4.2 | 4109.9 | 30.4 | 2133.4 | 15.8 | 145.5 | 2.1 | 4257.9 | 48.9 | 1064.7 | 11.0 | 35.4 | 0.1 | 6.0 | 0.1 | 25.6 | 0.3 | 41.0 | 0.2 | 10.0 | 0.2 |
| **G89** | **K0204** | INDIA | 560.1 | 0.6 | 3409.6 | 10.3 | 1852.1 | 6.8 | 133.5 | 1.7 | 3181.2 | 10.0 | 1450.3 | 6.5 | 19.9 | 0.0 | 6.0 | 0.0 | 41.8 | 0.1 | 35.0 | 0.2 | 14.1 | 0.2 |
| **G90** | **K616** | INDIA | 160.7 | 1.4 | 3812.2 | 27.1 | 1667.4 | 10.5 | 124.7 | 2.2 | 3818.6 | 14.0 | 1260.0 | 13.0 | 28.3 | 0.3 | 6.4 | 0.1 | 49.1 | 0.5 | 44.0 | 0.3 | 13.4 | 0.5 |
| **G91** | **K8020** | INDIA | 367.7 | 4.1 | 3595.3 | 48.5 | 1736.8 | 15.9 | 151.3 | 7.0 | 3703.5 | 70.5 | 1176.9 | 14.0 | 29.0 | 0.3 | 5.7 | 0.2 | 22.3 | 0.2 | 42.7 | 0.6 | 11.4 | 0.3 |
| **G92** | **K86** | INDIA | 161.3 | 1.5 | 4113.8 | 26.6 | 1931.7 | 7.4 | 138.8 | 3.1 | 3921 | 40.2 | 1212.5 | 8.6 | 29.0 | 0.1 | 5.3 | 0.1 | 28.1 | 0.1 | 41.0 | 0.2 | 12.2 | 0.2 |
| **G93** | **K88** | INDIA | 307.1 | 1.9 | 4113.8 | 43.9 | 1555.5 | 2.6 | 138.3 | 4.1 | 3440.6 | 7.6 | 1107.9 | 12.0 | 25.9 | 0.1 | 4.3 | 0.0 | 28.7 | 0.1 | 35.4 | 0.1 | 8.0 | 0.2 |
| **G94** | **K911** | INDIA | 140.0 | 1.3 | 4252.1 | 13.7 | 1965.0 | 6.2 | 156.5 | 5.0 | 4130.9 | 25.7 | 1297.0 | 8.4 | 31.7 | 0.3 | 7.8 | 0.1 | 30.3 | 0.3 | 39.7 | 0.3 | 12.3 | 0.2 |
| **G95** | **KALYANSONA** | INDIA | 250.9 | 1.5 | 3788.7 | 12.1 | 1604.6 | 10.6 | 120.6 | 2.1 | 3572.7 | 15.0 | 1067.4 | 6.6 | 23.9 | 0.3 | 3.3 | 0.0 | 18.8 | 0.1 | 38.3 | 0.1 | 9.8 | 0.1 |
| **G96** | **KLP306** | INDIA | 280.6 | 1.1 | 3414.4 | 26.6 | 1663.0 | 7.2 | 141.5 | 4.7 | 2870.8 | 21.4 | 1673.1 | 8.5 | 23.0 | 0.1 | 7.0 | 0.0 | 33.1 | 0.1 | 38.2 | 0.1 | 13.5 | 0.4 |
| **G97** | **KLP307** | INDIA | 562.3 | 0.2 | 3673.7 | 11.6 | 2042.6 | 5.4 | 154.6 | 3.4 | 3368.0 | 15.5 | 1451.4 | 21.0 | 26.2 | 0.0 | 7.0 | 0.0 | 30.3 | 0.1 | 32.9 | 0.2 | 13.0 | 0.3 |
| **G98** | **NAW1448** | INDIA | 527.5 | 3.9 | 3410.8 | 24.1 | 2000.9 | 12.8 | 154.0 | 4.1 | 3420.6 | 11.7 | 1395.8 | 2.7 | 32.2 | 0.5 | 7.2 | 0.2 | 40.8 | 0.3 | 38.8 | 0.4 | 13.4 | 0.6 |
| **G99** | **NW1076** | INDIA | 491.9 | 1.3 | 3960.3 | 17.1 | 1751.8 | 6.6 | 173.4 | 2.0 | 3795.7 | 6.2 | 1094.2 | 12.0 | 33.5 | 0.1 | 4.0 | 0.0 | 30.4 | 0.1 | 42.4 | 0.2 | 10.9 | 0.4 |
| **G100** | **NW2036** | INDIA | 197.0 | 0.4 | 3359.5 | 25.7 | 1520.3 | 5.8 | 127.1 | 1.6 | 3225.5 | 27.9 | 1061.4 | 7.7 | 23.4 | 0.1 | 3.9 | 0.1 | 25.1 | 0.1 | 39.0 | 0.2 | 13.5 | 0.2 |
| **G101** | **PBW550** | INDIA | 341.7 | 1.6 | 3780.9 | 20.3 | 1562.6 | 7.8 | 157.7 | 4.9 | 2734.8 | 9.8 | 1293.8 | 12.0 | 43.3 | 0.2 | 7.4 | 0.1 | 33.5 | 0.3 | 30.9 | 0.2 | 12.0 | 0.3 |
| **G102** | **RAJ1482** | INDIA | 531.1 | 1.8 | 3854.3 | 35.2 | 1876.9 | 6.0 | 186.6 | 5.4 | 3979.6 | 42.5 | 1115.6 | 2.9 | 31.1 | 0.2 | 4.3 | 0.0 | 29.3 | 0.1 | 51.7 | 0.2 | 10.3 | 0.4 |
| **G103** | **RAJ3072** | INDIA | 455.9 | 1.9 | 4005.0 | 31.4 | 1857.8 | 2.1 | 169.2 | 3.7 | 3762.8 | 11.7 | 1025.9 | 22.0 | 30.6 | 0.3 | 5.4 | 0.1 | 24.7 | 0.0 | 41.8 | 0.2 | 10.4 | 0.1 |
| **G104** | **RAJ3077** | INDIA | 149.0 | 1.7 | 3678.3 | 46.0 | 1523.2 | 5.5 | 109.0 | 6.3 | 3247.4 | 33.2 | 976.3 | 22.0 | 21.2 | 0.1 | 4.3 | 0.1 | 20.2 | 0.1 | 35.5 | 0.2 | 11.5 | 0.3 |
| **G105** | **RAJ3777** | INDIA | 352.1 | 1.5 | 3597.3 | 23.4 | 1635.8 | 14.5 | 162.6 | 2.0 | 3256.5 | 16.6 | 1075.3 | 8.0 | 23.3 | 0.1 | 5.3 | 0.1 | 29.3 | 0.2 | 40.4 | 0.1 | 10.9 | 0.4 |
| **G106** | **RAJ4027** | INDIA | 208.3 | 0.8 | 4909.4 | 46.7 | 1762.4 | 4.9 | 167.3 | 7.4 | 4015.6 | 10.2 | 1382.0 | 12.0 | 32.7 | 0.3 | 9.7 | 0.2 | 33.4 | 0.3 | 30.3 | 0.1 | 14.1 | 0.4 |
| **G107** | **RAJ4037** | INDIA | 256.9 | 1.6 | 3635.6 | 33.8 | 1706.3 | 6.7 | 150.3 | 3.0 | 3301.4 | 31.8 | 1115.1 | 17.0 | 29.3 | 0.3 | 6.1 | 0.1 | 28.7 | 0.3 | 39.5 | 0.3 | 11.6 | 0.2 |
| **G108** | **RAJ4120** | INDIA | 150.5 | 1.6 | 4306.1 | 32.8 | 1716.0 | 8.2 | 174.4 | 5.4 | 3651.8 | 22.0 | 1135.0 | 5.7 | 26.6 | 0.2 | 4.0 | 0.0 | 30.8 | 0.2 | 38.4 | 0.2 | 11.6 | 0.3 |
| **G109** | **SAW327** | INDIA | 448.4 | 3.7 | 3993.4 | 9.7 | 1739.4 | 10.8 | 129.8 | 1.2 | 3826.6 | 16.5 | 1091.8 | 5.0 | 31.8 | 0.4 | 5.1 | 0.0 | 32 | 0.1 | 38.4 | 0.3 | 11.4 | 0.3 |
| **G110** | **SAW337** | INDIA | 350.7 | 2.9 | 3717.6 | 28.3 | 1899.5 | 11.9 | 157.3 | 5.4 | 3898.6 | 23.2 | 1229.7 | 23.0 | 32.9 | 0.2 | 5.4 | 0.1 | 20.9 | 0.1 | 34.4 | 0.2 | 11.2 | 0.3 |
| **G111** | **SAW94** | INDIA | 514.0 | 3.2 | 4416.2 | 38.7 | 1939.4 | 14.1 | 138.4 | 2.6 | 4348.7 | 25.0 | 1180.5 | 16.0 | 36.4 | 0.1 | 5.4 | 0.1 | 26.4 | 0.1 | 39.9 | 0.2 | 11.7 | 0.3 |
| **G112** | **SONALIKA** | INDIA | 316.1 | 3.1 | 3913.1 | 54.3 | 1556.7 | 11.2 | 141.7 | 7.7 | 3132.4 | 23.9 | 888.3 | 5.1 | 23.3 | 0.3 | 4.4 | 0.1 | 18.2 | 0.2 | 31.8 | 0.3 | 9.5 | 0.3 |
| **G113** | **UP2338** | INDIA | 212.0 | 0.7 | 3962.9 | 38.8 | 1767.3 | 4.0 | 129.2 | 4.3 | 3931.0 | 32.9 | 1218.7 | 16.0 | 32.0 | 0.2 | 4.8 | 0.1 | 29.0 | 0.1 | 47.7 | 0.0 | 11.2 | 0.2 |
| **G114** | **UP2511** | INDIA | 489.1 | 3.0 | 4926.1 | 23.4 | 2060.5 | 10.7 | 179.1 | 3.9 | 3989.8 | 31.1 | 1437.6 | 8.1 | 42.9 | 0.5 | 8.7 | 0.1 | 37.6 | 0.2 | 30.5 | 0.3 | 13.2 | 0.3 |
| **G115** | **UP2525** | INDIA | 170.1 | 1.2 | 3999.8 | 59.1 | 1743.4 | 15.9 | 126.7 | 6.3 | 4039.3 | 5.1 | 1189.3 | 2.6 | 26.7 | 0.2 | 4.6 | 0.1 | 21.9 | 0.3 | 33.9 | 0.3 | 8.9 | 0.1 |
| **G116** | **UP2696** | INDIA | 510.9 | 2.7 | 3300.7 | 38.4 | 1816.3 | 1.5 | 150.2 | 6.1 | 3243.0 | 25.3 | 1441.7 | 8.0 | 40.9 | 0.3 | 7.3 | 0.1 | 46.9 | 0.9 | 25.5 | 0.3 | 16.2 | 0.6 |
| **G117** | **VEERI** | INDIA | 319.4 | 0.9 | 4531.2 | 29.3 | 1710.3 | 3.5 | 235.4 | 3.8 | 4010.9 | 41.1 | 1108.2 | 11.0 | 35.6 | 0.4 | 10.7 | 0.1 | 34.2 | 0.1 | 35.9 | 0.2 | 8.7 | 0.3 |
| **G118** | **VL832** | INDIA | 182.0 | 2.1 | 4112.6 | 29.2 | 1534.4 | 8.5 | 156.0 | 4.0 | 3692.2 | 35.9 | 1240.7 | 11.0 | 22.1 | 0.2 | 4.9 | 0.1 | 26.1 | 0.3 | 28.4 | 0.1 | 11.1 | 0.4 |
| **G119** | **WR1381** | INDIA | 427.3 | 2.7 | 2986.2 | 12.7 | 1836.3 | 11.2 | 137.6 | 2.9 | 2912.7 | 11.6 | 1482.3 | 32.0 | 19.1 | 0.1 | 4.9 | 0.1 | 33.8 | 0.2 | 31.4 | 0.0 | 13.1 | 0.4 |
| **G120** | **WR1408** | INDIA | 517.6 | 1.8 | 4017.3 | 17.1 | 1790.2 | 12.3 | 117.4 | 2.3 | 2982.0 | 29.2 | 1413.0 | 4.5 | 22.6 | 0.2 | 6.7 | 0.1 | 38.2 | 0.2 | 34.0 | 0.1 | 9.1 | 0.2 |
| **G121** | **WR1421** | INDIA | 228.4 | 2.0 | 3336.7 | 30.1 | 1712.8 | 7.3 | 135.3 | 3.5 | 2696.7 | 12.3 | 1572.2 | 15.0 | 23.7 | 0.2 | 6.6 | 0.1 | 28.1 | 0.2 | 26.6 | 0.1 | 14.3 | 0.2 |
| **G122** | **KARAHAN_99** | TURKEY | 137.2 | 1.7 | 3281.9 | 41.5 | 1770.4 | 1.6 | 136.1 | 5.7 | 2684.4 | 10.6 | 1623.8 | 7.9 | 17.6 | 0.0 | 9.1 | 0.2 | 39.1 | 0.1 | 18.1 | 0.1 | 16.4 | 0.3 |
| **G123** | **EKIZ** | TURKEY | 214.4 | 1.4 | 2934.9 | 23.6 | 1469.8 | 5.4 | 107.7 | 4.8 | 1983.3 | 7.1 | 1251 | 8.2 | 10.8 | 0.1 | 5.1 | 0.0 | 22.8 | 0.1 | 39.3 | 0.2 | 13.2 | 0.3 |
| **G124** | **AT_052_K2** | TURKEY | 295.4 | 0.9 | 3547.3 | 26.9 | 1770.5 | 16.0 | 109.3 | 4.0 | 3286.2 | 33.1 | 1226.9 | 15.0 | 35.9 | 0.3 | 15.6 | 0.1 | 23.3 | 0.2 | 65.6 | 0.2 | 10.3 | 0.2 |
| **G125** | **KAMCI** | TURKEY | 223.2 | 1.4 | 3813.6 | 46.4 | 1500.1 | 4.8 | 130.6 | 3.7 | 3016.2 | 15.5 | 1213.1 | 8.9 | 27.0 | 0.0 | 6.6 | 0.1 | 43.4 | 0.3 | 49.8 | 0.2 | 13.2 | 0.4 |
| **G126** | **SARIBAS** | TURKEY | 359.5 | 1.9 | 3374.9 | 20.8 | 1463.7 | 9.7 | 126.6 | 2.9 | 2304.7 | 8.1 | 1126.0 | 12.0 | 22.8 | 0.0 | 7.4 | 0.1 | 37.9 | 0.2 | 45.3 | 0.2 | 10.1 | 0.4 |
| **G127** | **DAGDAS** | TURKEY | 204.0 | 2.0 | 3540.6 | 40.0 | 1581.8 | 3.0 | 110.3 | 5.3 | 2565.4 | 22.1 | 1403.3 | 19.0 | 13.0 | 0.1 | 8.8 | 0.1 | 29.0 | 0.1 | 40.8 | 0.2 | 13.1 | 0.4 |
| **G128** | **ADANA** | TURKEY | 478.8 | 3.4 | 3492.6 | 26.5 | 1663.4 | 88.7 | 134.6 | 4.0 | 2885.1 | 28.0 | 1268.5 | 8.5 | 31.4 | 0.1 | 7.1 | 0.1 | 37.7 | 0.2 | 36.3 | 0.2 | 12.4 | 0.2 |
| **G129** | **TOSUNBEY** | TURKEY | 199.2 | 0.6 | 2834.2 | 20.4 | 1712.0 | 3.7 | 125.4 | 4.5 | 2573.3 | 6.8 | 1323.9 | 15.0 | 11.2 | 0.1 | 6.2 | 0.0 | 35.4 | 0.2 | 36.6 | 0.2 | 14.0 | 0.4 |
| **G130** | **MURAT** | TURKEY | 387.3 | 1.7 | 3193.0 | 23.4 | 1519.1 | 7.1 | 105.4 | 4.0 | 2730.4 | 3.9 | 1447.1 | 8.7 | 39.3 | 0.2 | 7.1 | 0.2 | 42.6 | 0.2 | 62.8 | 0.3 | 13 | 0.2 |
| **G131** | **ATILLA** | TURKEY | 221.5 | 0.5 | 3094.4 | 9.0 | 1497.6 | 4.8 | 103.4 | 1.5 | 2497.0 | 7.7 | 1381.2 | 7.6 | 40.0 | 0.1 | 9.0 | 0.1 | 34.7 | 0.1 | 50.2 | 0.1 | 14.6 | 0.4 |
| **G132** | **AHMETAGA** | TURKEY | 367.2 | 2.7 | 3164.3 | 18.1 | 1635.4 | 10.1 | 116.0 | 2.1 | 2373.2 | 26.1 | 1156.5 | 19.0 | 17.6 | 0.1 | 7.8 | 0.0 | 49.7 | 0.4 | 19.4 | 0.1 | 13.9 | 0.2 |
| **G133** | **DEMIR** | TURKEY | 527.0 | 0.5 | 2860.4 | 32.5 | 1678.0 | 11.9 | 173.8 | 5.1 | 2417.7 | 4.0 | 1464.1 | 5.7 | 25.0 | 0.1 | 5.3 | 0.0 | 23.5 | 0.1 | 58.3 | 0.2 | 16.3 | 0.3 |
| **G134** | **BAGCI** | TURKEY | 120.6 | 1.2 | 4583.7 | 22.5 | 1918.0 | 16.8 | 123.8 | 3.1 | 3472.5 | 9.9 | 1414.0 | 12.0 | 10.7 | 0.2 | 5.4 | 0.0 | 30.5 | 0.2 | 19.4 | 0.1 | 15.0 | 0.3 |
| **G135** | **BAYRAKTAR** | TURKEY | 201.2 | 1.4 | 3117.3 | 14.4 | 1560.8 | 7.3 | 483.1 | 6.6 | 2892.7 | 13.3 | 1052.2 | 8.6 | 17.2 | 0.1 | 6.2 | 0.1 | 30.5 | 0.3 | 29.8 | 0.1 | 13.2 | 0.2 |
| **G136** | **SEVAL** | TURKEY | 313.7 | 2.1 | 3235.3 | 10.3 | 1466.6 | 15.9 | 109.5 | 1.6 | 2034.8 | 6.4 | 1259.7 | 3.6 | 20.2 | 0.1 | 7.2 | 0.1 | 32.5 | 0.0 | 35.0 | 0.1 | 13.8 | 0.2 |
| **G137** | **KENANBEY** | TURKEY | 366.6 | 2.7 | 3176.9 | 20.1 | 1661.2 | 11.0 | 142.2 | 2.6 | 2194.9 | 10.0 | 1041.4 | 3.8 | 18.3 | 0.1 | 6.2 | 0.1 | 21.0 | 0.1 | 27.2 | 0.1 | 10.2 | 0.5 |
| **G138** | **BEZOSTAJA** | TURKEY | 274.5 | 1.5 | 3196.2 | 6.0 | 1427.7 | 7.5 | 104.8 | 1.7 | 2297.7 | 0.9 | 1338.0 | 17.0 | 32.1 | 0.1 | 7.2 | 0.1 | 40.3 | 0.1 | 58.3 | 0.3 | 13.8 | 0.3 |
| **G139** | **GUN91** | TURKEY | 298.6 | 1.5 | 3092.8 | 10.7 | 1451.3 | 7.1 | 113.0 | 1.8 | 2289.5 | 7.9 | 1050.7 | 5.2 | 27.5 | 0.0 | 6.8 | 0.0 | 37.1 | 0.2 | 48.0 | 0.2 | 10.8 | 0.2 |
| **G140** | **KONYA** | TURKEY | 321.6 | 2.4 | 3030.6 | 19.1 | 1534.5 | 4.0 | 97.6 | 2.2 | 2193.2 | 8.0 | 1164.3 | 15.0 | 16.0 | 0.1 | 5.2 | 0.1 | 43.5 | 0.1 | 23.0 | 0.1 | 14.2 | 0.2 |
| **G141** | **AKBUGDAY** | TURKEY | 229.0 | 0.1 | 3412.0 | 29.6 | 1422.9 | 13.8 | 103.5 | 2.9 | 2390.8 | 7.4 | 1450.1 | 28.0 | 21.5 | 0.1 | 6.9 | 0.1 | 28.4 | 0.1 | 33.3 | 0.1 | 17.3 | 0.5 |
| **G142** | **GEREK79** | TURKEY | 356.5 | 0.7 | 4597.5 | 35.9 | 1733.5 | 6.0 | 136.4 | 2.1 | 3263.8 | 16.6 | 1343.4 | 15.0 | 26.4 | 0.1 | 7.4 | 0.1 | 23.9 | 0.2 | 38.4 | 0.1 | 13.7 | 0.2 |
| **G143** | **KIRAC66** | TURKEY | 253.3 | 2.9 | 3396.5 | 12.8 | 1542.8 | 9.6 | 97.6 | 2.2 | 2895.6 | 34.5 | 1098.6 | 6.9 | 14.0 | 0.2 | 6.0 | 0.0 | 15.7 | 0.2 | 36.0 | 0.3 | 11.6 | 0.3 |
| **G144** | **ESER** | TURKEY | 424.4 | 1.3 | 2841.4 | 11.2 | 1700.7 | 4.3 | 101.8 | 1.3 | 2147.9 | 6.1 | 1157.4 | 14.0 | 16.1 | 0.1 | 5.8 | 0.0 | 30.2 | 0.1 | 29.7 | 0.1 | 11.7 | 0.4 |
| **G145** | **SONMEZ** | TURKEY | 107.0 | 1.2 | 3013.6 | 20.0 | 1266.8 | 2.6 | 109.2 | 2.6 | 1926.7 | 5.4 | 1103.8 | 3.6 | 27.4 | 0.1 | 7.3 | 0.1 | 22.5 | 0.2 | 34.0 | 0.2 | 10.1 | 0.4 |
| **G146** | **HARMANKAYA** | TURKEY | 226.5 | 0.3 | 3038.9 | 51.8 | 1570.4 | 3.9 | 121.0 | 7.8 | 1912.4 | 21.9 | 1318.8 | 8.9 | 14.1 | 0.0 | 7.7 | 0.1 | 28.0 | 0.2 | 21.8 | 0.2 | 12.8 | 0.1 |
| **G147** | **KINACI** | TURKEY | 199.3 | 1.9 | 3610.5 | 22.6 | 1337.9 | 12.3 | 114.6 | 2.6 | 2595.3 | 23.7 | 929.1 | 9.4 | 30.6 | 0.3 | 4.6 | 0.1 | 37.7 | 0.1 | 40.9 | 0.1 | 10.4 | 0.2 |
| **G148** | **YUREGIR** | TURKEY | 141.1 | 0.9 | 3761.8 | 9.4 | 1537.0 | 18.5 | 309.9 | 3.0 | 3235.3 | 31.5 | 1047.4 | 14.0 | 18.4 | 0.1 | 4.8 | 0.1 | 32.5 | 0.2 | 23.6 | 0.1 | 10.4 | 0.2 |
| **G149** | **ALTAY** | TURKEY | 222.9 | 0.7 | 3995.6 | 6.1 | 1768.9 | 10.7 | 139.5 | 1.6 | 3145.2 | 37.0 | 1390.5 | 14.0 | 26.2 | 0.1 | 6.2 | 0.0 | 29.6 | 0.1 | 29.7 | 0.1 | 13.8 | 0.2 |
| **G150** | **LUTFIBEY** | TURKEY | 104.3 | 1.0 | 2852.4 | 20.9 | 1431.4 | 11.8 | 81.3 | 1.9 | 1775.0 | 6.8 | 975.6 | 3.7 | 12.6 | 0.0 | 4.5 | 0.0 | 21.9 | 0.2 | 23.6 | 0.0 | 10.6 | 0.2 |
