# Supplementary material for: Assessment of genetic variability for grain nutrients from diverse regions: potential for wheat improvement
Source: Springerplus. 2016 Nov 3;5(1):1912. doi: 10.1186/s40064-016-3586-2 (PMC5095102; doi:10.1186/s40064-016-3586-2)
Supplement: Supplementary file 2 — Additional file 2. Table S2. Loadings of the elemental and protein variables of the first three Principal Components (PC) for 121 Indian and 29 Turkish bread wheat genotypes. [file 40064_2016_3586_MOESM2_ESM.docx]

**Table S2.** Loadings of the elemental and protein variables of the first three Principal Components (PC) for 121 Indian and 29 Turkish bread wheat genotypes.

| **Variable** | **PC1** | **PC2** | **PC3** |
| --- | --- | --- | --- |
| Ca | 0.365 | -0.006 | -0.168 |
| K | 0.251 | -0.296 | 0.364 |
| Mg | 0.469 | -0.090 | 0.076 |
| Na | 0.201 | -0.125 | 0.403 |
| P | 0.402 | -0.373 | 0.078 |
| S | 0.292 | 0.461 | 0.120 |
| Zn | 0.432 | -0.041 | -0.196 |
| Cu | 0.175 | 0.395 | -0.119 |
| Fe | 0.173 | 0.345 | -0.096 |
| Mn | 0.184 | -0.078 | -0.703 |
| GPC | 0.124 | 0.504 | 0.307 |
| Total Variance (%) | 30.5 | 19.0 | 11.4 |
| Cumulative Variance (%) | 30.5 | 49.5 | 60.8 |
