# Supplementary material for: Assessment of genetic variability for grain nutrients from diverse regions: potential for wheat improvement
Source: Springerplus. 2016 Nov 3;5(1):1912. doi: 10.1186/s40064-016-3586-2 (PMC5095102; doi:10.1186/s40064-016-3586-2)
Supplement: Supplementary file 3 — Additional file 3. Table S3. Working conditions and specifications of ICP-AES in this experiment. [file 40064_2016_3586_MOESM3_ESM.docx]

**Table S3 .** Working conditions and specifications of ICP-AES in this experiment.

| **ICP-AES** | **Properties** |
| --- | --- |
| RF power | 0.7-1.8 Kw (1.2-1.3 kW for axial) |
| Auxilary gas flow rate (Ar) | 1.5 |
| Plasma gas flow rate (Pr) | 10.5-15 L/min (radial), 15 “(axial)” |
| Copy time | 3 s (max. 100 s) |
| Copy and reading time | 1-5 s (max. 60 s) |
| Viewing height | 5-12 mm |
